# SUMO and KSHV Replication

**DOI:** 10.3390/cancers6041905

**Published:** 2014-09-29

**Authors:** Pei-Ching Chang, Hsing-Jien Kung

**Affiliations:** 1 Institute of Microbiology and Immunology, National Yang-Ming University, Taipei 112, Taiwan; E-Mail: pcchang@ym.edu.tw; 2 Institute for Translational Medicine, College of Medical Science and Technology, Taipei Medical University, Taipei 110, Taiwan; 3 Department of Biochemistry and Molecular Medicine, University of California, Davis, CA 95616, USA; 4 UC Davis Cancer Center, University of California, Davis, CA 95616, USA; 5 Division of Molecular and Genomic Medicine, National Health Research Institutes, 35 Keyan Road, Zhunan, Miaoli County 35053, Taiwan

**Keywords:** KSHV, SUMO, epigenetic, PML-NB, interferon

## Abstract

Small Ubiquitin-related MOdifier (SUMO) modification was initially identified as a reversible post-translational modification that affects the regulation of diverse cellular processes, including signal transduction, protein trafficking, chromosome segregation, and DNA repair. Increasing evidence suggests that the SUMO system also plays an important role in regulating chromatin organization and transcription. It is thus not surprising that double-stranded DNA viruses, such as Kaposi’s sarcoma-associated herpesvirus (KSHV), have exploited SUMO modification as a means of modulating viral chromatin remodeling during the latent-lytic switch. In addition, SUMO regulation allows the disassembly and assembly of promyelocytic leukemia protein-nuclear bodies (PML-NBs), an intrinsic antiviral host defense, during the viral replication cycle. Overcoming PML-NB-mediated cellular intrinsic immunity is essential to allow the initial transcription and replication of the herpesvirus genome after *de novo* infection. As a consequence, KSHV has evolved a way as to produce multiple SUMO regulatory viral proteins to modulate the cellular SUMO environment in a dynamic way during its life cycle. Remarkably, KSHV encodes one gene product (K-bZIP) with SUMO-ligase activities and one gene product (K-Rta) that exhibits SUMO-targeting ubiquitin ligase (STUbL) activity. In addition, at least two viral products are sumoylated that have functional importance. Furthermore, sumoylation can be modulated by other viral gene products, such as the viral protein kinase Orf36. Interference with the sumoylation of specific viral targets represents a potential therapeutic strategy when treating KSHV, as well as other oncogenic herpesviruses. Here, we summarize the different ways KSHV exploits and manipulates the cellular SUMO system and explore the multi-faceted functions of SUMO during KSHV’s life cycle and pathogenesis.

## 1. Introduction

### 1.1. SUMO as a Signal Transducer

**Figure 1:**
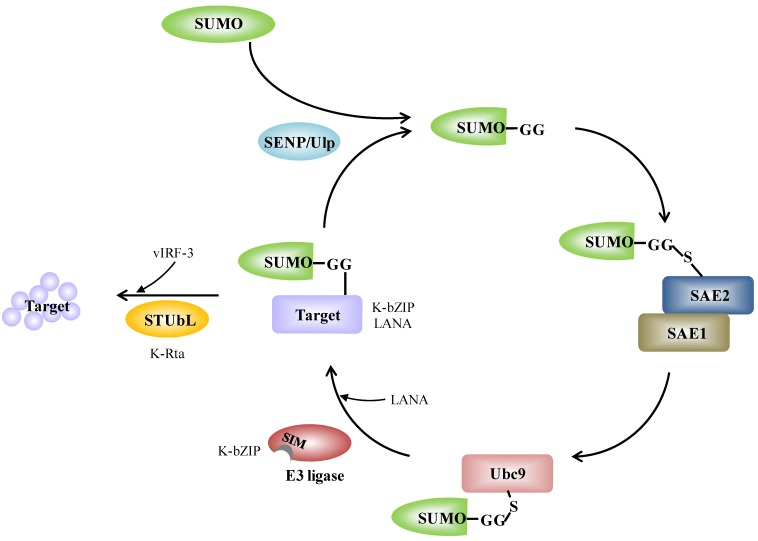
The SUMO conjugation enzyme cascade pathway and its links with KSHV. SUMO precursors are cleaved at the c-terminus by SENP and this reveals a GG motif. Mature SUMO is activated by conjugation to the SUMO E1 activating enzyme (SAE1/SAE2). Activated SUMO is transferred to the SUMO E2 conjugating enzyme (Ubc9). Ubc9 itself is able to catalyze SUMO conjugation to the lysine residue of a target proteins. SUMO E3 ligases are dispensable, but increase target specificity. SUMO is removed from the target by SENP, which cleaves SUMO from the substrate and releases free SUMO; alternatively, STUbL targets the sumoylated protein for degradation. The scheme also illustrates the proteins of KSHV and the corresponding components in SUMO modification system. K-bZIP itself is a SUMO E3 ligase and LANA acts as a SUMO E3 ligase. K-Rta itself is a STUbL and vIRF-3 acts as a STUbL. Detailed information is given in the text.

SUMO has been found in all eukaryotes. There are three major protein conjugating SUMO isoforms in mammals. One is SUMO-1, and then there are SUMO-2 and SUMO-3, which are highly homologous (they are often referred to as SUMO-2/3). Like ubiquitination, SUMO conjugation occurs through an enzyme cascade. SUMO precursors are processed by SUMO-specific proteases (SENPs). Mature SUMO is activated by the SUMO E1 activating enzyme (SAE1/SAE2), transferred to the SUMO E2 conjugating enzyme (Ubc9), and then ligated to a lysine residue within SUMO consensus sequence inside the target protein (Figure 1). Although SUMO E3 ligase does not seem to be essential for the *in vitro* sumoylation reaction, it enhances the efficiency and specificity of SUMO attachment inside the cells (Figure 1) (Reviewed in [1]). A large number of proteins have been identified that are selectively modified by SUMO-1 or by SUMO-2/3. However, the mechanisms underlying the selective nature of the SUMO isoforms remains incompletely understood.

Similar to the Src homology 2 (SH2) domains and phosphotyrosine binding (PTB) domains that mediate selective protein-protein interaction with phosphorylated tyrosine, the SUMO-interacting motif (SIM) mediates the recognition of SUMO modification in both a paralog-specific and -non-specific manner [2,3]. Via SIM and SUMO moieties, SUMO modification serves as a platform to recruit specific proteins. In addition, similar to phosphorylation, sumoylation is a reversible process, and SUMO can be removed by SENPs. In mammals, there are six SENPs that vary in SUMO paralog specificity [4]. Taken as a whole, two potential mechanisms for paralog-specific SUMO modification have been described. One is based on paralog-specific sumoylation that is mediated by the SIM [5,6] and the other is related to the protection of specific SUMO paralogs from desumoylation by SENPs [7]. SUMO E3 ligases are likely to be what determines paralog-specific sumoylation. However, none of the cellular SUMO E3 ligases, including the PIAS (protein inhibitor of activated STAT) family, RanBP2 (Ran-binding protein 2), and Pc2, have been shown to have distinct selectivity toward a particular SUMO paralog, although some SUMO E3 ligases do display SUMO paralog-specificity toward certain targets.

Due to the involvement of SUMO in almost all cellular aspects, it is not surprising that viruses have evolved many different pathways to exploit the host SUMO system. In most cases, viruses target the SUMO pathway by encoding proteins that interact with various components of the pathway in order to modulate their enzyme activities. For instance, the avian adenovirus, chicken embryo lethal orphan (CELO), encodes Gam1 that inactivates the SUMO E1 activating enzyme [8]. Human adenovirus serotype 5 (HAdV5) encodes E1A that interacts with Ubc9 and interferes with polysumoylation [9]. Many viral proteins are modified by SUMO (reviewed elsewhere [10,11,12]). For example, the E1B-55K protein of HAdV5 [13], the Zta (BZLF1) and Rta proteins of Epstein-Barr virus (EBV) [14,15], and the K-bZIP and LANA proteins of KSHV are all sumoylated proteins [16,17]. In addition to targeting cellular SUMO machinery, a few viruses encode viral proteins that have activities analogous to sumoylation enzymes including both SUMO E3 ligase and SUMO-targeted ubiquitin ligase (STUbL). For example, the first identified viral SUMO E3 ligase, K-bZIP, is encoded by KSHV and shows SUMO-2/3 selectivity [18]. The protein E1B-55K from HAdV5 is another viral SUMO E3 ligase that shows specificity toward SUMO-1 [19,20]. In terms of activity similar to STUbL, the first example of a viral protein with STUbL-like properties was the ICP0 protein from herpes simplex virus type 1 (HSV-1) [21]. Another example is K-Rta of KSHV [22]. Thus far, there has been no report describing viral proteins that have activities analogous to SUMO E1 activating enzyme (SAE1/SAE2), SUMO E2 conjugating enzyme (Ubc9), or SENP.

### 1.2. KSHV and SUMO

KSHV, also known as human herpesvirus type 8 (HHV-8), is a γ-herpesvirus associated with Kaposi’s sarcoma (KS), primary effusion lymphomas (PEL) and multicentric Castlemen’s disease (MCD) [23]. It primarily infects endothelial and B cells. It is one of the seven recognized human cancer viruses [24]. Like all herpesviruses, the KSHV life cycle is divided into two distinct phases, the lytic phase and the latent phase. Based on expression kinetics, KSHV genes can be categorized into immediate-early (IE), early (E), late (L), and latent genes. IE genes are transcribed first after lytic reactivation, which is followed by E genes and then by L genes. IE genes encode regulatory proteins that control the expression of viral and cellular genes. The E genes initiate viral genome replication and the L genes encode most of the viral structural proteins of the viral capsid [25,26]. Latent genes are those transcribed in latently infected cells and are often involved in viral episome maintenance, replication and the transformation of host cells [27]. While KSHV genomic DNA is naked in the virion, viral DNA forms a chromatin-like structure in latently infected cells. The decoration of the viral episome with activating and repressive histone modifications allows latent genes to be expressed during viral latency, and early lytic genes to be maintained in a silent but poised state during the latent phase [28,29], thus being ready to be turned on by extrinsic factors during lytic reactivation. KSHV is able to maintain a tightly controlled latent infection in the majority of the tumor cells, but a small subset of cells often undergo spontaneous lytic replication [30,31]. Establishing latency enables KSHV to sustain a life-long infection and to escape immune surveillance [32,33]. Lytic replication not only allows the spread of KSHV infection, but also is a prerequisite for sustaining the population of latently infected cells and allowing KSHV pathogenesis [34,35,36]. Recently, it has been found by several laboratories that the KSHV genome displays quite different epigenetic landscapes during the lytic and latent states, which suggests that chromatin remodeling is involved in the transition from latency to the lytic phase [28,29,37,38]. Sumoylation is known to affect chromatin structure during transcription, DNA repair and replication, and therefore the relevance of sumoylation to KSHV reactivation and latency entry is expected. In this review, we will describe how KSHV encodes proteins that are involved in the SUMO network and explain how they potentially have an impact on viral replication.

### 1.3. KSHV, SUMO and Antiviral Immunity

In addition to chromatin remodeling, SUMO plays another important role in the herpesvirus life cycle, namely in the assembly and disassembly of PML-NBs or ND-10 (nuclear domain-10). PML-NBs are discrete subnuclear structures that contain multiple cellular proteins, including PML, Sp100, hDaxx, and ATRX. It has long been postulated that PML-NBs function as an intrinsic antiviral defense by repressing viral transcription and replication [39]. Disruption of PML-NBs is essential for efficient infection and the lytic replication of a number of DNA viruses, including herpesviruses [21,40,41,42]. Moreover, a recent study using a mouse model suggested that PML is also able to repress the establishment and maintenance of latent infection by herpesvirus [43]. As a result, relocalization and/or the degradation of PML would seem to be a key step in herpesvirus replication and this process often involves sumoylation and ubiquitination.

The PML-NB is a SUMO storage warehouse for the nucleus and it is here that a large number of sumoylated proteins congregate. Sumoylation of PML is a prerequisite for recruitment of Daxx, Sp100, and SUMO paralogs during PML-NB formation [44]. De-sumoylation or the degradation of PML results in the disassembly of the PML-NBs [45]. A key enzyme involved in the disassembly of PML-NBs is RNF4, which is the cellular STUbL [46,47]. In this review we describe how KSHV utilizes viral STUbL-like activity to degrade PML.

Moreover, KSHV targets SUMO because SUMO is capable of modulating interferon-related antiviral responses. Invading viruses are recognized by Toll-like receptors (TLRs), a type of pathogen recognition receptor (PRRs), and this then results in the production of type I interferon (IFN) and the subsequent activation of the host innate immune response. Transcriptional induction of the type I IFN genes is mainly regulated by the IFN regulatory factor-3 (IRF-3) and IFN regulatory factor-7 (IRF-7). Sumoylation of IRF-3 and IRF-7 brings about the negative regulation of type I IFN expression [48]. As a result, it is possible for viruses to exploit sumoylation in order to suppress the type I IFN-mediated anti-viral response. For example, EBV latent membrane protein 1 (LMP1) and Ebola Zaire virus VP35 induce sumoylation of IRF-7, which limits its transactivation ability to elicit the immune response [49,50]. Vesicular stomatitis virus (VSV) infection triggers sumoylation of both IRF-3 and IRF-7 to attenuate the interferon production [48]. Taken together, there are at least three reasons why KSHV exploits various cellular SUMO activities. The first is the latency to lytic switch, the second is the disassembly of PML-NBs and the third is related to the anti-interferon responses.

## 2. KSHV Proteins are Involved in Modulating Various SUMO Activities

### 2.1. The KSHV IE Gene K-Rta (KSHV-Replication and Transcriptional Activator)

**Figure 2:**
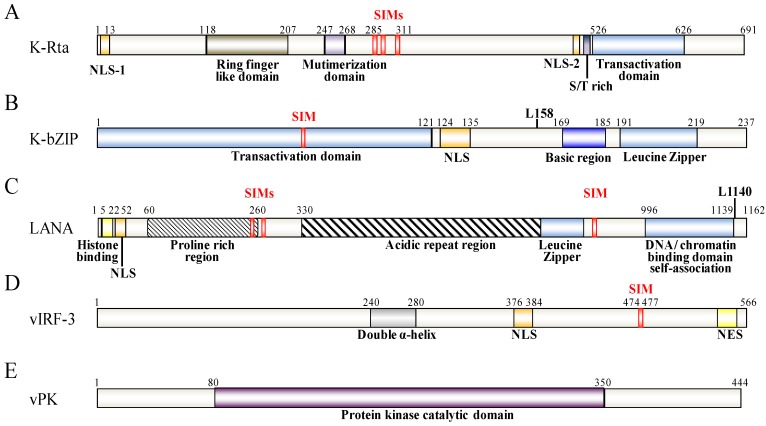
Schematic representation of KSHV K-Rta (**A**), K-bZIP (**B**), LANA (**C**), vIRF-3 (**D**) and vPK (**E**). The functional domains of these proteins are indicated below the schematic. The SIM and the SUMO lysine (L) sites are depicted above the protein structure.

K-Rta is the first KSHV protein expressed after *de novo* infection or during the reactivation process. It is a potent transcription factor that is necessary and sufficient to trigger the full lytic phase of KSHV. Concomitant with its ability to induce lytic replication and break the virus latency, K-Rta expression remodels the viral chromatin and disassembles PML-NBs [22,51]. K-Rta’s exceptional transactivation ability is partly due to its ability to assemble active transcriptional cofactors and chromatin remodeling proteins and partly due to its ability to manipulate the sumoylation of viral and cellular proteins [22]. K-Rta has been shown to have ubiquitin ligase activity [52,53] and has the ability to associate with the cellular ubiquitin ligase RAUL to degrade IRF-3 and IRF-7 [54]. Recently, it has been further shown that K-Rta acts as a Ub E3 ligase, preferring sumoylated substrates and behaving like a SIM domain containing STUbL [22] (Figure 2A). Similar to RNF4, K-Rta contains tandem SIM domains and prefers to bind and degrade SUMO-2/3 polymer forms. Both the SIM and Ring-finger like domains are necessary for the STUbL activity of K-Rta. The STUbL activity of K-Rta is essential for K-Rta transactivation and KSHV replication. Importantly, by degrading SUMO-2/3 and sumoylated cellular proteins, K-Rta is able to create a “SUMO-light” cellular environment [22]. The protein also preferentially degrades its antagonist K-bZIP and sumoylated PML (see below). Overexpressing SUMO-2 and the SUMO E2 conjugating enzyme Ubc9 partially reverses the “SUMO-light” phenotype caused by K-Rta expression. They also attenuate K-Rta induced KSHV gene expression and reactivation, which indicates that the modulation of cellular SUMO activity plays a key role in controlling KSHV replication. Likewise, SUMO represses host gene expression during viral reactivation. Knockdown of SUMO-2/3 results in the induction of host genes transcription during K-Rta mediated KSHV reactivation [55]. SUMO-2/3 enrichment within the cellular gene promoter region during KSHV reactivation negatively regulates constitutively expressed genes and prevents them from activating during the viral lytic cycle. The ability of K-Rta to disassemble the PML-NB using its own STUbL activity is shared by HSV ICP0 [21]. CMV IE1 and VZV IE also have the ability to degrade the PML-NB, although it is not clear whether they are STUbLs themselves or are able to interact with the cellular Ub E3 ligase in order to effect PML degradation. EBV Rta, a K-Rta homolog, is not a STUbL, but can induce PML-NB degradation [56]; furthermore, it can be sumoylated by RanBP and is recognized by RNF4 for degradation [57]. Thus, it appears that IE proteins of herpesviruses all have the ability to interact with the cellular SUMO network, but their various modes of action are distinct.

### 2.2. The KSHV IE Gene K-bZIP (KSHV Basic Leucine Zipper Protein)

K-bZIP is another KSHV IE lytic cycle gene product and is encoded by K8, which is immediately downstream from the K-Rta locus on the viral genome. It is a strong transcriptional repressor that directly associates with K-Rta and is able to attenuate K-Rta-mediated transactivation activity [58]. K-bZIP is sumoylated primarily at a specific lysine residue (Lys-158) and this SUMO modification is essential for it to function as a transcriptional repressor [17]. In additional to being modified by SUMO, K-bZIP is a SIM domain containing SUMO E3 ligase that shows higher specificity toward SUMO-2/3 [18] (Figure 2B).

**Figure 3:**
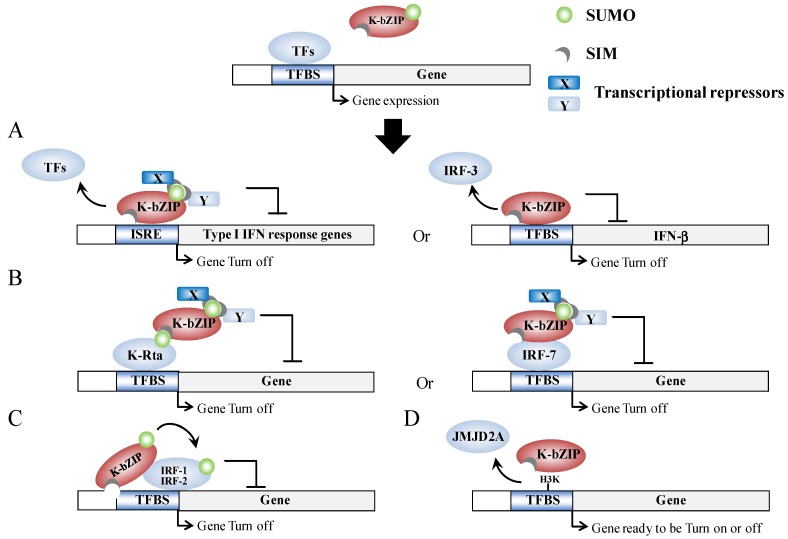
Model outlining epigenetic regulation by K-bZIP. (**A**) K-bZIP is able to directly compete for TFBS binding with TFs in the promoter of target genes in a sumoylation-dependent (left) or in a sumoylation-independent (right) manner; (**B**) K-bZIP is able to interact with TFs in a SUMO-dependent (left) or in a SUMO-independent (right) manner, which in turn inhibits gene expression via sumoylation-dependent recruitment of transcriptional repressors; (**C**) Sumoylation of TFs by K-bZIP in a SIM-dependent manner; (**D**) K-bZIP is able to directly interact with and compete with histone modification enzyme activity in SUMO-independent manner.

Host gene shut-off is a phenotype commonly associated with herpesvirus infection [59,60]. The suppression of cellular gene expression is important, as it results in the shut-down of host innate immune response genes and allows the economical use of cellular resource for its own replication. For KSHV, there are several gene products that are able to potentially function during host shut-off. The examples include, the SOX protein, which is encoded by Orf37 (RNase/DNase) [59,61], and the K-bZIP [38]. Genome-wide profiling of the chromatin binding patterns of SUMO paralogs has shown that SUMO-2/3 is significantly increased during KSHV reactivation and this enrichment occurs on the promoters of a group of immune-related genes that show no elevation of expression during KSHV reactivation [55]. Moreover, the annotation of SUMO peaks in relation to putative transcription factor binding sites (TFBS) obtained from Transfac Matrix Database shows that potential SUMO-2/3 targeting transcription factors (TFs) are significantly increased from 22 to 86 during KSHV reactivation. Interestingly, three interferon regulatory transcription factors, IRF-7, IRF-1, and IRF-2, have been identified as 4th, 5th, and 6th most potent SUMO-2/3 target TFs, respectively, after KSHV reactivation. This suggests that K-bZIP catalyzes the sumoylation of these interferon response factors in order to attenuate their activity, which is consistent with the finding that K-bZIP enhances SUMO modification of IRF-1 and IRF-2 and that it interacts with IRF-7 [55]. In line with this, K-bZIP was found to target interferon (IFN) pathways through both SUMO-dependent and -independent mechanisms. For example, K-bZIP has also been found to inhibit interferon-α (IFN-α) signaling in a sumoylation dependent manner [62]. On the other hand, K-bZIP can also inhibit IFN-β signaling by impeding IRF-3 binding to the IFN-β promoter in a SUMO-independent manner [63]. Moreover, K-bZIP is able to directly interact with a histone demethylase, JMJD2A, and this binding blocks its demethylase activity as well as the access to its substrate H3K9me3. Inhibition of JMJD2A by K-bZIP results in the global increase of the repressive marker, H3K9me3, which may in part account for the general host gene shut-off observed in K-bZIP overexpressing cells [38]. Overall, as summarized in Figure 3, K-bZIP would seem to use both SUMO-dependent and SUMO-independent strategies to modulate viral and host gene expression.

While a proportion of K-bZIPs colocalize within the PML-NB, this alone does not appear to cause PML-NB’s dispersion, and, in fact, this may increase the SUMO content in the PML-NB. However, it is conceivable that, in the presence of K-Rta, PML or other proteins are sumoylated by K-bZIP and thus can be preferentially degraded by K-Rta. We postulate that, depending on the ratio of K-bZIP over K-Rta at a given time during viral infection, the fraction of cellular sumoylated proteins including PML may increase or decrease in a dynamic manner. The SUMO-2/3 proteins deposited on the viral genome clearly increase during the late phase of viral reactivation, which coincides with the increased expression of K-bZIP and the cessation of K-Rta expression. This is consistent with the view that K-bZIP has the ability to increase sumoylation and that this may help condense the viral chromosome facilitating entry into latency [64,65]. That said, at least in an *in vitro* cell culture system, a K-bZIP deletion mutant is still able to enter latency, albeit at a much lower efficiency [65]. This suggests that enhanced sumoylation by K-bZIP may not be an absolutely requirement for entry into latency, and such sumoylation may possibly also be carried out by a cellular SUMO E3 ligase under certain conditions. A similar model has been proposed for the role of sumoylation with respect to the Zta protein of EBV [66] in entry into latency. Like K-bZIP, EBV Zta is sumoylated and this sumoylation, which decreases its transactivation ability, is negatively regulated by the viral protein kinase BGLF4. Unlike K-bZIP, EBV’s Zta is not a SUMO ligase, but it does have the ability to disassemble the PML-NB. It has been suggested that this involves a competition of EBV Zta with PML with respect to sumoylation [14]. In addition to the KSHV lytic cycle IE gene, K-Rta, a KSHV lytic cycle late gene, Orf75, has also been found to target the PML-NB. Orf75 is a tegument protein that induces the disappearance of ATRX, a PML-NB component, which results in the relocalization of the PML-NB [67]. These findings suggest that KSHV uses a variety of strategies to antagonize the antiviral replication effects of PML-NBs.

In summary, K-bZIP is a general transcriptional repressor. K-bZIP overexpression has been found to result in a global repression of gene expression [38]. Similarly, enrichment of SUMO modification across the host genome during KSHV reactivation is associated with a shut-off of transcription. The concomitant rise of K-bZIP expression and SUMO-2/3 binding across the KSHV genome after KSHV reactivation suggests that the SUMO E3 ligase activity of K-bZIP may help the virus to enter latency, a phase in which most viral genes are silenced.

### 2.3. The KSHV Latent Gene LANA (Latency-Associated Nuclear Antigen)

LANA is the dominant viral latent protein that is essential for the establishment of latency and the maintenance of the viral episome [68]. LANA is a DNA binding protein that, firstly, binds to the latent origins within the terminal repeats (TR) to mediate viral DNA replication [69,70,71], secondly, tethers the KSHV episomes to the host chromosome helping their proper segregation during cell division [68] and, thirdly, serves as a transcriptional activator or repressor, depending on the protein’s associated cofactors and the targeted promoter [72,73,74,75]. LANA has also been implicated in the transformation of target cells by sequestering the transcriptional and proapoptotic activities of p53 [76,77].

**Figure 4:**
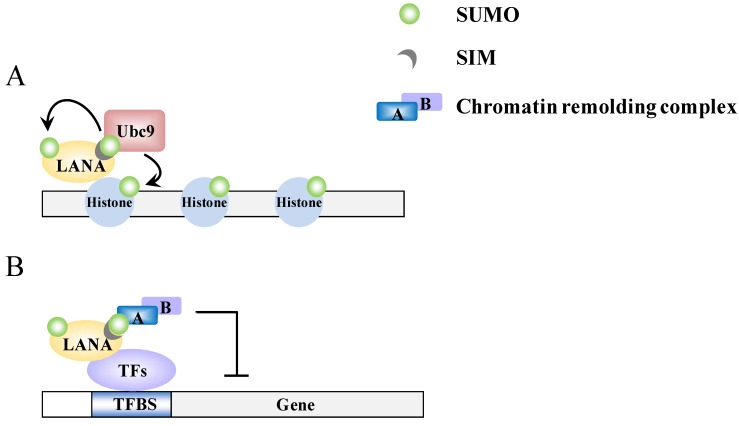
A model of epigenetic regulation by LANA. (**A**) LANA recruits Ubc9 to specific genomic regions and promotes sumoylation of histone proteins; (**B**) The SIM-dependent recruitment of SUMO-modified chromatin-remodeling complexes by LANA.

Recently, LANA has been found to have a strong SUMO connection. LANA can be sumoylated and contains both N-terminal and C-terminal SIM domains. There are two SIMs (SIM1 and SIM2) within the N-terminal domain and these preferentially bind SUMO-2 [16,51] (Figure 2C). Cai *et al.* found that these SIMs are essential for various processes that LANA is involved in. Specifically this involves the recruitment of various SUMO modified chromatin remodeling proteins and transcription factors, such as KAP-1 and HIF-1α, respectively, to their binding sites (Figure 4B) and sumoylated of LANA at lysine 1140 to maintain the KSHV episome, to silence K-Rta, and to block viral lytic replication [16]. In essence, LANA, through its ability to bind SUMO and to be sumoylated, recruits repressor proteins such as KAP-1 that help to maintain the latent genome in a silenced state. This is consistent with our previous finding that KAP-1 is recruited to and widely-spread across the latent viral chromatin, and that the protein dissociates upon reactivation [78]. Interestingly, in a recent review of Campbell and Izumiya, they provide some preliminary findings suggesting that LANA may possesses SUMO ligase activity toward histones H2A and H2B, and that this requires the C-terminal SIM domain [51]. They further showed that LANA decorates the latent chromosome in a way matching the SUMO pattern, which suggests either that LANA itself is sumoylated or that the histone H2A and H2B associated with LANA is sumoylated. Alternatively, KAP-1, which associates with LANA, is known to be sumoylated; this association may also contribute to the SUMO distribution that is seen across the viral chromatin. Most likely, it is a combination of all these factors that result in the latent viral genome being heavily “coated” with SUMO (Figure 4A). Taken together, the findings imply that SUMO modification assists LANA-mediated chromatin condensation in both a global and a gene-specific manner (Figure 4).

### 2.4. The KSHV Latent Gene vIRF-3/LANA2 (Viral Interferon Regulatory Factor-3/Latency-Associated Nuclear Antigen 2)

vIRF-3 is another KSHV latent cycle gene that is encoded by K10.5. This protein is expressed exclusively in KSHV-infected B cells and is required for the survival of KSHV-infected primary effusion B-cell lymphoma (PEL) [79]. vIRF-3 possesses a SIM motif at the protein’s C-terminus [80] and itself can be sumoylated at multiple sites [80,81] (Figure 2D). In addition to directly interacting with and being conjugated by SUMO, vIRF-3 is also capable of inhibiting the sumoylation of proteins that it interacts with, such as the retinoblastoma family of pocket proteins, pRb, p107 and p130 [82]. Inhibition of pRb sumoylation by vIRF-3 blocks pRb-induced cell growth arrest [82]. In addition, while not a SUMO E3 ligase itself, vIRF-3 has been shown to be capable of dispersing PML-NBs by increasing the sumoylation and ubiquitination of PML in a SIM-dependent manner [72,81]. Although the detailed mechanism has yet to be elucidated, it is possible that vIRF-3 upregulates Ubc9 or RNF4. Alternatively, the protein may increase the affinity between these molecules and the PML-NB. vIRF-3 is a latent protein and thus its functionality in terms of degrading PML in the context of lytic replication is not clear. However, vIRF-3 is shown to increase in the expression of survivin, an anti-apoptotic molecule repressed by PML, and thus vIRF-3-mediated PML degradation may contribute to the transformation properties of KSHV [72].

### 2.5. The KSHV Lytic Gene vPK (Viral Protein Kinase)

vPK is an early lytic gene encoded by Orf36. It is a serine/threonine protein kinase that is localized in the nucleus and can be autophosphorylated [83]. K-bZIP has been shown to harbor two phosphorylation sites at residue 111 and 167, vPK interacts with KSHV K-bZIP and phosphorylates it at threonine 111. Phosphorylation of K-bZIP by vPK decreases the sumoylation level of K-bZIP and reduces the transcription repression activity of K-bZIP [84]. This regulatory phenomenon has also been observed with a cellular chromatin remodeling molecule, KAP-1, which is targeted by vPK. Sumoylation of KAP-1 is essential for the association of KAP-1 with heterochromatin and mediation of gene repression. Phosphorylation of KAP-1 by vPK causes a loss of KAP-1 sumoylation and this results in a consequential decrease in KAP-1 mediated chromatin condensation and a reduction in the repression of gene expression [78]. Phosphorylation-dependent antagonism of sumoylation by vPK has also revealed another level of sumoylation regulation by KSHV. Interestingly, the EBV vPK homolog, BGLF4, is a SIM containing protein and this molecule has been found to be able to negatively modulate the sumoylation of BZLF1 via phosphorylation, which occurs in a SIM-dependent manner [66,85]. Thus, the viral protein kinases of the γ-herpesvirus, while not sumoylated themselves, are intimately involved in the modulation of sumoylation, targeting both viral and host proteins. These findings underscore the importance of sumoylation to viral replication.

## 3. The SUMO Dynamics during KSHV Reactivation

Genome-wide profiling has shown that there is distinct dynamic to the chromatin-binding patterns of SUMO paralogs during viral reactivation. The SUMO-1 and SUMO-2/3 binding patterns are very similar in non-reactivated control cells. However, during KSHV reactivation, SUMO-2/3 binding, but not SUMO-1, is significantly increased in the human genome. This enrichment is focused on the promoters region that contain primary regulatory elements such as TFBSs. Annotation of SUMO sites with putative TFBSs identified potential SUMOylated TFs. It shows that potential SUMO-1 target TFs are decreased, while SUMO-2/3 targeting TFs are significantly increased during viral reactivation. Interestingly, three IRFs have been identified as top 10 most potent SUMO-2/3 target TFs after KSHV reactivation. Knockdown of SUMO-2/3 results in a higher induction of most those IRFs-regulated genes analyzed during viral reactivation. These findings suggest that one (or a few) of the KSHV lytic proteins are able to catalyze the sumoylation of these IRFs and attenuate their activity. Expression profiling revealed that these SUMO-2/3 targeted highly transcribed genes show no expression changes during viral reactivation. Gene ontology (GO) analysis showed that these genes are involved in cellular immune responses. Both TF identification and GO analysis indicates that SUMO-2/3 preferentially targets immune-related genes in order to make sure that the expression of these genes remains unaltered during viral reactivation [55].

Sumoylation is a post-translational modification that is both rapid and reversible. Modulation of sumoylation has a profound effect on protein-protein interactions and chromatin assembly. A common property of herpesviruses is that these processes are involved in the biphasic euchromatin-to-heterochromatin transition and this helps to bring about the rapid lytic/latency switch. Most studies have indicated that many chromatin-remodeling proteins are SUMO modified, and such SUMO modified proteins can recruit more SIM containing chromatin-remodeling enzymes that help to regulate chromatin structure. The rapid and reversible character of SUMO modification is able to efficiently affect the local dynamic of euchromatization and heterochromatization. Therefore, this makes sumoylation an interesting process whereby herpesvirus is able to regulate their rapid entry into latency and their rapid reactivation as part of their life cycle.

## 4. Conclusions and Future Perspectives

**Figure 5:**
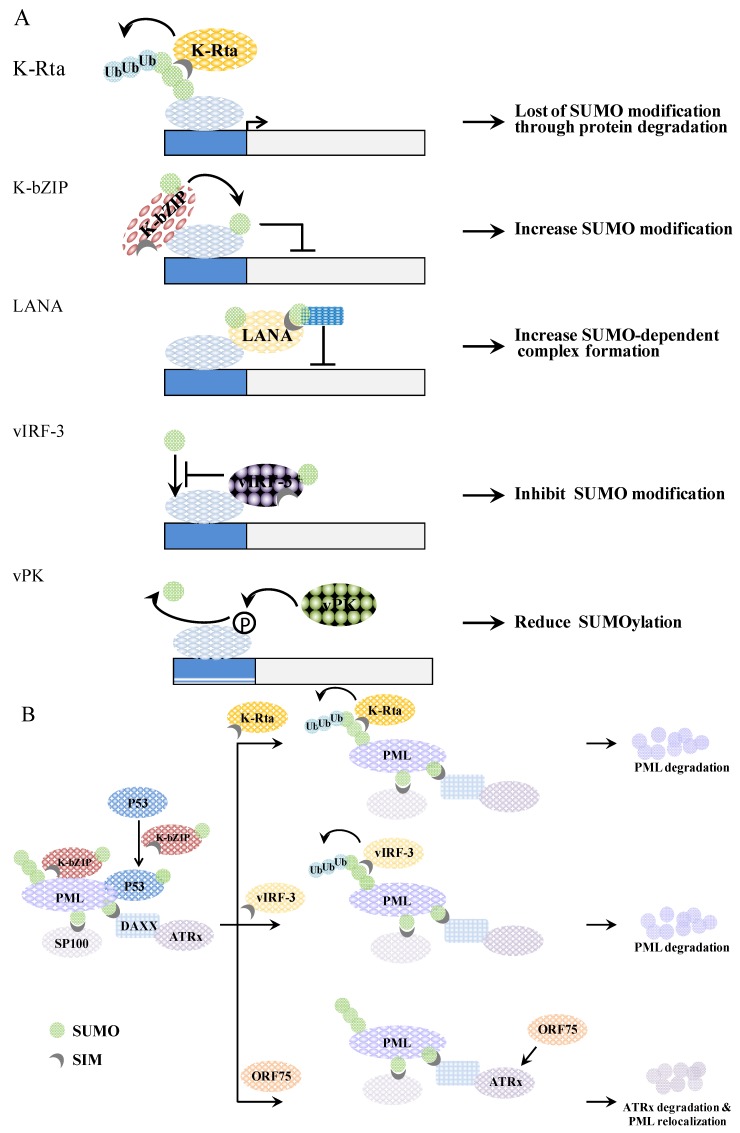
A schematic representation of genome (**A**) and PML-NB (**B**) regulation by KSHV viral proteins. (**A**) K-Rta induces SUMO-dependent ubiquitin-mediated proteasome degradation of SUMO modified proteins. K-bZIP mediates SUMOylation of interacting proteins. LANA elicits a recruitment of SUMO-modified protein. vIRF-3 inhibits the SUMOylation of interacting proteins. Phosphorylation of target protein by vPK causes reduction of SUMO modification; (**B**) K-bZIP colocalizes with PML in the PML-NB. K-bZIP sumoylates p53 and recruits p53 to the PML-NB. K-Rta functions as a STUbL by ubiquitinating SUMO chains on PML, which then induces the degradation of these sumoylated PML molecules. vIRF-3 promotes a SUMO dependent ubiquitylation of PML and this induces PML degradation and PML-NB disruption in a SIM-dependent manner. Orf75 induces ATRX degradation, which causes relocalization of the PML-NB.

Sumoylation, like phosphorylation, is involved in nearly all cellular processes and viruses have evolved different strategies to manipulate the SUMO process to their own advantage. For herpesviruses, sumoylation is important to at least three steps of viral replication and pathogenesis. These are: firstly, modulation of the viral chromatin during the latent and lytic states; secondly, the disassembly of PML-NBs; and thirdly, the attenuation of interferon responses. To these ends, KSHV encodes at least five proteins that are involved in these processes. They are as follows. Firstly, K-Rta, a lytic gene product with STUbL activity, which preferentially catalyzes the degradation of SUMO-2/3 and sumoylated proteins including PML [22]. Secondly, K-bZIP, a lytic gene product with SUMO E3 ligase activity [18], which is a strong transcriptional repressor and catalyzes the sumoylation of host chromatin remodeling proteins, as well as those involved in innate immunity. Thirdly, LANA, a latent protein with histone H2B sumoylation activity, which is the pivotal protein involved in latency maintenance and malignant transformation. Fourthly, vIRF-3, a latent gene product that promotes the SUMO-dependent ubiquitination and degradation of PML and the PML-NBs [80,81]. Finally, vPK, a lytic gene product and a serine/threonine protein kinase, which is involved in the modulation of the phosphorylation-dependent sumoylation of K-bZIP and KAP-1. It is interesting that KSHV encodes as many as five gene products that are able to manipulate the SUMO pathway. Three of them, and another KSHV gene product, Orf75, disrupt PML-NBs (Figure 5), which underscores the importance of overcoming the antiviral activity of PML-NBs in order to ensure herpesvirus survival. Perhaps the most striking finding of KSHV is that, with its limited genome capacity, the virus encodes at least one SUMO E3 ligase and one STUbL of its own. This is interesting, considering that only a very few STUbLs (one or two) and SUMO E3 ligases (five) are encoded by the whole host genome, and suggests that SUMO plays a special role in the life cycle of KSHV. Taken together, disrupting the SUMO conjugating pathway may be one means to break the balance of KSHV lytic and latent cycle, which is important to persistent infection. Therefore, a derangement of sumoylation may help the clearance of herpesviruses from chronically infected cells and help to improve current therapy. This makes SUMO a potential target for antiviral therapy. Moreover, the presence of different SUMO paralogs in mammals and the SUMO paralog specificity of KSHV encoded SUMO E3 ligase leads to a potential hypothesis whereby SUMO paralogs may play a variety of quite different roles in viral life cycle. Elucidating the preferential usage of SUMO paralogs in herpesviruses will help to improve the specificity of any SUMO-targeted antiviral therapies.
